# Letter to the editor: Does Dicer Expression Affect shRNA processing?

**DOI:** 10.4137/grsb.s2551

**Published:** 2009-06-19

**Authors:** Neil Senzer, Donald Rao, John Nemunaitis

**Affiliations:** 1 Mary Crowley Cancer Research Centers, Dallas, TX; 2 Gradalis, Inc., Carrollton, TX. Email:jnemunaitis@marycrowley.org

**Keywords:** dicer expression, Drosha mRNA, shRNA, cancer

## Abstract

Elevated Dicer and Drosha mRNA levels have been documented across a range of tumor types (including ovarian carcinoma) by a number of investigators without any demonstrable correlation with patient survival nor evidence of interference with shRNA processing. A recent publication by Merritt et al. (NEJM 359(25):2641-50, 2008) reporting their findings in patients with ovarian carcinoma reach opposite conclusions. Further study will be needed to resolve this issue.

Using Affymetrix GeneChip Microarray, we recently documented 0.2 to 2,145 fold Dicer mRNA over expression normalized to comparator tissue in 26 patients; primarily lung, breast and colon. In addition, we and others have demonstrated effective shRNA mediated silencing in cells with low Dicer expression. Although a global downregulation of miRNA (also dependent on Dicer mediated biogenesis) in cancer was initially reported using the Luminex bead-based profiling assay,^1^ no significant differences in Dicer or Drosha mRNA were detected in that study (n = 334 samples) nor has there since been any clinically based definitive linkage between Dicer mRNA levels and miRNA signatures. In a comparison of ovarian serous adenocarcinoma to normal ovarian surface epithelium, Drosha was upregulated 2.7 fold and Dicer 1.6 fold^2^ without any association of Dicer expression with either disease-free or overall survival. Similarly, despite confirming miRNA downregulation in late stage ovarian cancer, Zhang found no significant differences in Dicer or Drosha mRNA between early versus late stage disease or between epithelial ovarian carcinoma cell lines and immortalized primary cultured human ovarian surface epithelium.^3^ Nor was a correlation found between Dicer (or Drosha) expression levels and survival. Moreover, even though Dicer expression has been shown to be low in both monocytic cell lines and monocytes derived from healthy donors,^4^ shRNA is functionally effective in CD34+ derived macrophages as well as in monocytic cell lines and monocyte-derived macrophages (see ref. 56 and 57 in ref. 4). The authors claim that the low levels of Dicer in these cell types are sufficient to support miRNA biogenesis. The “low” Dicer expressing cell lines may, therefore, have slower knockdown kinetics for shRNA rather than being less effective. If not, then miRNA levels in shRNA treated cells, a more biologically relevant indicator, should be lower. Yet Merritt et al. recently reported decreased Dicer and Drosha mRNA and expression in both cell lines and tumor samples from patients with ovarian cancer.^5^ Sixty percent of specimens had decreased normalized Dicer mRNA. Furthermore, low Dicer levels were found to be a predictor of reduced disease-specific survival in multivariate analysis. Of particular interest was the finding that, compared to siRNA mediated silencing of the galectin-3 gene, poor silencing was achieved with shRNA in ovarian cancer cell lines with low versus high Dicer expression. Further evaluation of these contradicting results will be required.
